# Development of Delivery Systems Enhances the Potency of Cell-Based HIV-1 Therapeutic Vaccine Candidates

**DOI:** 10.1155/2021/5538348

**Published:** 2021-04-20

**Authors:** Amin Hadi, Abbas Rastgoo, Maryam Eskandarian, Nooshin Haghighipour, Azam Bolhassani

**Affiliations:** ^1^Cellular and Molecular Research Center, Yasuj University of Medical Sciences, Yasuj, Iran; ^2^School of Mechanical Engineering, University of Tehran, Tehran, Iran; ^3^Department of Immunology, Tarbiat Modares University, Tehran, Iran; ^4^National Cell Bank of Iran, Pasteur Institute of Iran, Tehran, Iran; ^5^Department of Hepatitis and AIDs, Pasteur Institute of Iran, Tehran, Iran

## Abstract

An effective therapeutic vaccine to eradicate HIV-1 infection does not exist yet. Among different vaccination strategies, cell-based vaccines could achieve in clinical trials. Cell viability and low nucleic acid expression are the problems related to dendritic cells (DCs) and mesenchymal stem cells (MSCs), which are transfected with plasmid DNA. Thus, novel *in vitro* strategies are needed to improve DNA transfection into these cells. The recent study assessed immune responses generated by MSCs and DCs, which were derived from mouse bone marrow and modified with Nef antigen using novel methods in mice. For this purpose, an excellent gene transfection approach by mechanical methods was used. Our data revealed that the transfection efficacy of Nef DNA into the immature MSCs and DCs was improved by the combination of chemical and mechanical (causing equiaxial cyclic stretch) approaches. Also, chemical transfection performed two times with 48-hour intervals further increased gene expression in both cells. The groups immunized with Nef DC prime/rNef protein boost and then Nef MSC prime/rNef protein boost were able to stimulate high levels of IFN-*γ*, IgG2b, IgG2a, and Granzyme B directed toward Th1 responses in mice. Furthermore, the mesenchymal or dendritic cell-based immunizations were more effective compared to protein immunization for enhancement of the Nef-specific T-cell responses in mice. Hence, the use of chemical reagent and mechanical loading simultaneously can be an excellent method in delivering cargoes into DCs and MSCs. Moreover, DC- and MSC-based immunizations can be considered as promising approaches for protection against HIV-1 infections.

## 1. Introduction

The success of combination antiretroviral therapy (cART) has resulted in the reduction of mortality associated with human immunodeficiency virus (HIV). However, the existing cART strategies are not able to remove the virus from infected individuals. Thus, it will be needed for a considerably effective HIV therapeutic vaccine to induce cellular immunity and cause virally infected cells to death [1, 2]. Tat and Nef are important HIV-1 regulatory proteins, which improve viral replication and downregulate the expression of MHC class I molecules, respectively. These proteins were considered to be targets for the development of the HIV vaccine [3].

The mesenchymal stem cells (MSCs) derived from bone marrow can transfer therapeutic proteins [4]. MSCs were employed to produce antimicrobial prophylactic or anticancer therapeutic vaccines. However, it was required to efficiently deliver and express transgenes in human MSCs [5]. On the other hand, potent antigen-presenting cells (APCs) such as dendritic cells (DCs) were used to prepare therapeutic vaccines [6]. It was indicated that DC-based therapeutic HIV-1 vaccines increase HIV-specific T-cell responses leading to a variety of clinical trials. Despite the fact that these vaccines were well-tolerated and safe, virologic and immunologic responses were inconsistent [6]. Several strategies were designed to load DCs with foreign antigens, such as electroporation and lipofection, and could increase immunogenicity in mice, but these strategies had some problems such as toxicity and low DNA expression *in vivo* [7].

Generally, effective intracellular delivery methods are required to transfect DNA into DCs and also MSCs. Tian et al. [8] showed that mechanotransduction is an approach for changing mechanical stimuli into electrochemical signals; however, the exact mechanism of cellular responses to local mechanical signals is indefinite. Mechanotransduction was generated in the cells using a variety of stimuli such as cyclic shear stress [9, 10], cyclic stretch [11, 12], and hydrostatic pressure [13–15]. The studies showed that mechanical stresses effectively transport genes into the cells [16]. In addition, DNA-repeated transfections (48-hour interval for 168 hours) could deliver DNA in high levels without cytotoxicity *in vitro* and *in vivo* as compared to a single use [2].

In this study, a mechanical bioreactor causing equiaxial cyclic stretch was used for gene transfection such as pEGFP-Nef and pEGFP-N1 transfection into MSCs and DCs. The transfection efficiency of a commercial chemical reagent such as Lipofectamine or TurboFect was assessed individually and in combination with the mechanical approach. In the present study, immune responses induced by DCs and MSCs expressing HIV-1 Nef antigen were assessed. Also, these responses were compared with the results of Nef protein immunization in BALB/c mice.

## 2. Materials and Methods

### 2.1. Generation of the Recombinant pEGFP-Nef Construct

DNA Extraction Midi Kit (Qiagen) was used for preparing the eukaryotic expression vector (pEGFP-N1) which harbors the full length of the HIV-1 Nef gene (pEGFP-Nef, Nef sequence from HIV-1 vector pNL4-3, accession no. AF324493.2) following the manufacturer's instructions. Also, it was applied to prepare the empty pEGFP-N1 (this vector encodes a variant of wild-type GFP which has been optimized for brighter fluorescence and higher expression in mammalian cells) on a large scale, which is considered as a positive control. The NanoDrop spectrophotometry was utilized for quantification. The presence of the Nef gene in the pEGFP vector was approved by digestion with restriction enzymes as previously reported [17].

### 2.2. Generation of the Recombinant HIV-1 Nef Protein

The expression of recombinant HIV-1 Nef protein (rNef) was performed in *Escherichia coli* Rosetta strain [18], and the affinity chromatography by employing a Ni-NTA agarose column was used for protein purification based on the manufacturer's instructions (Qiagen). As a result of monitoring by the LAL assay (QCL-1000, Lonza), the endotoxin contamination was shown to be lower than 0.5 EU per milligram protein. After dialysis of the purified protein, its amount was evaluated by NanoDrop spectrophotometry and stored at -70°C.

### 2.3. Generation of Dendritic Cells (DCs)

As previously described, the extraction of DCs was performed from the bone marrow of male BALB/c mice provided by the animal center of Pasteur Institute of Iran (IPI) [19]. DCs were identified by staining with conjugated antibodies for CD11c, CD40, CD86, and MHCII markers [20]. FACS analysis was conducted on a FACScan flow cytometer (Becton Dickinson). Then, mechanical and chemical methods were used to transfect the DCs with pEGFP-Nef; these methods were discussed in the following sections. The modified DCs (~1.0 × 10^6^ cells) were employed in the vaccination regimens.

### 2.4. Isolation of Mesenchymal Stem Cells (MSCs)

Isolation of MSCs was done from the bone marrow of male BALB/c mice provided by the animal center of IPI, as previously reported [19, 21]. For transfection, the third passage of MSCs in complete DMEM (Sigma) including 10% heat-inactivated FBS was utilized. Flow cytometry analysis was used to identify the MSCs by CD90.2 and CD45 surface markers [19].

### 2.5. Transfection Using TurboFect or Lipofectamine Commercial Reagents

Lipofectamine 2000 (cationic lipid, Invitrogen) and TurboFect (cationic polymer, Fermentas) transfection reagents were employed to deliver pEGFP-Nef into DCs (1 × 10^5^ cell/well) and MSCs (2 × 10^5^ cell/well) in a 24-well plate. A FACSCalibur flow cytometer (Partec) was used to quantify the transfection efficiency at 72 hours posttransfection. The negative and positive controls were the untransfected cells and the transfected cells with pEGFP-N1 (Clontech, USA), respectively, [22].

### 2.6. Transfection Using a Mechanical Loading

**Figure 1:**
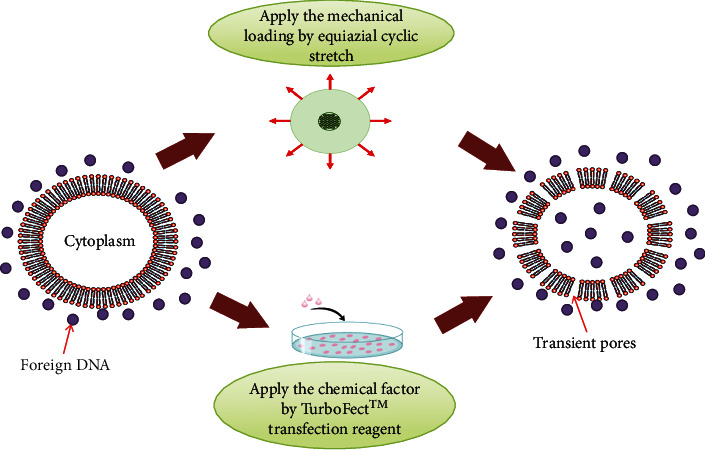
The schematic of the transfection method by the equiaxial cyclic stretch and chemical reagent.

**Figure 2:**
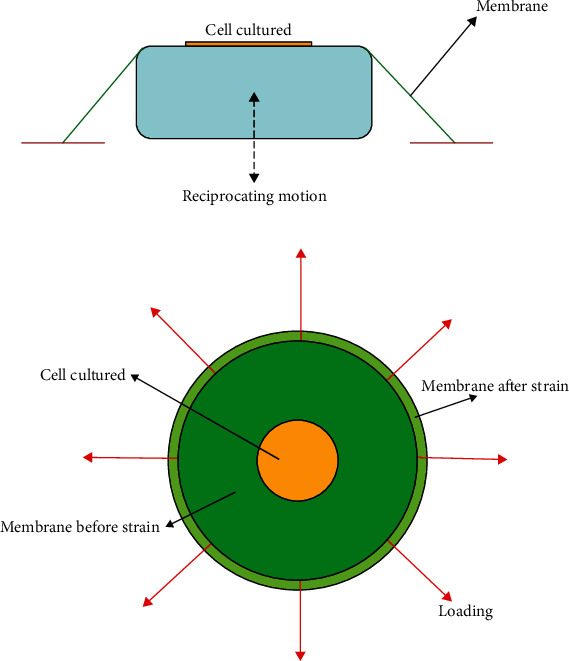
The schematic of an equiaxial cyclic bioreactor: (a) front view; (b) top view.

Transfection of MSC and DC cells using mechanical loading was done according to our previous studies on TC-1 tumor cells [16]. In summary, the DC and MSC cells were seeded, respectively, at densities of 100000 and 200000 cells on the center of the medical-grade silicone membrane and incubated for 3 hours. The collagen type I coating was used on the membrane for adhesion of DCs to the silicon membrane. An equiaxial cyclic stretch bioreactor made in IPI was employed to evaluate whether the mechanical loading affects the cells [16, 23]. The cells were cultured on the central part of the membrane and incubated overnight to perform the experiment; Figure 1 shows their schematic model. Transfection used equiaxial cyclic stretch. Various designs that apply uniaxial cyclic strain to cells cultured on a suitable elastic membrane or soft tissue and simulation of the internal mechanical environment of the body have been investigated, but each design has its limitations. An equiaxial cyclic stretch bioreactor in the National Cell Bank of Iran had been designed to apply a uniaxial cyclic stretch. In this step, mechanical loading was used. The range of applied strain was 5 to 10 percent, at a time interval of 1 hour and a frequency of 1 Hz according to previous research [16]. It should be noted that the test conditions must first be determined for any mechanical load to achieve the best transfection efficiency without causing any damage to the cell. After unloading and separating the membrane from the equiaxial cyclic stretch bioreactor, the membrane containing the loaded cell, the transfection operation should be performed. The schematic of an equiaxial bioreactor can be shown in Figure 2. As previously reported in our studies, the placement of loaded cultured cells was done in the proximity of the plasmid DNA without using any chemical transfection reagent to evaluate whether the mechanical loading affects the transfection rate [16]. Furthermore, the mechanical bioreactor was used to load the cells cultured on silicone membranes before chemical transfection for the simultaneous treatment by utilizing the mechanical loading and chemical reagent. Afterward, plasmid DNAs complexed with Lipofectamine or TurboFect were added to the medium and the cells were incubated at 37°C in a 5% CO_2_ incubator, as reported in our previous studies [16]. A FACSCalibur flow cytometer (Partec) was used to quantify the transfection efficiency at 72 h posttransfection.

### 2.7. Mouse Immunization

Five-seven-week-old inbred BALB/c female mice were housed and maintained following the guidelines of IPI (national guideline) for scientific purposes (code 925). As indicated in Table 1, different regimens were used to immunize three mice thrice with a two-week interval. Montanide ISA720 was employed to emulsify recombinant Nef protein at the ratio of 70 : 30 (*v*/*v*, oil: aqueous phase). Nef protein was injected at a concentration of 10 *μ*g based on our previous studies for induction of effective immune responses.

### 2.8. Assessment of Humoral Immune Responses

At 28 days after the second booster, the mice were bled from retroorbital (after anesthesia by utilizing intraperitoneal injection of ketamine (87.5 mg/kg)/xylazine (12.5 mg/kg) cocktail: 0.1 ml/20 g mouse). Subsequently, the sera were pooled for each group. The indirect ELISA was employed for evaluation of the production of goat anti-mouse IgG1, IgG2b, IgG2a, and total IgG antibodies (Sigma) [19]. The recombinant Nef protein was the coated antigen (~5 *μ*g/ml).

### 2.9. Measurement of Cytokines and Granzyme B

Granzyme B and cytokines were assessed as previously described [19]. In summary, at 28 days after the second booster, the random sacrification of mice from each group was performed after anesthesia. The spleens were removed, and the red blood cell-depleted splenocytes (2 × 10^6^ cells/ml) were cultured for 72 h in 48-well plates (Nunc, Germany) in the presence of 5 *μ*g/ml of the recombinant Nef protein or 5 *μ*g/ml of concanavalin A (as positive control). It should be mentioned that the splenocytes were pooled for each group before culture. According to the manufacturer's instructions, a DuoSet ELISA system (R&D Systems) was used to assess the levels of IL-10, IL-5, and IFN-*γ* cytokines by the sandwich-based ELISA method; hence, the supernatants were harvested to carry out the assessment. In order to assess the Granzyme B, the P815 target cells (T) were seeded in triplicate into 96-well U-bottomed plates (2 × 10^4^ cells/well) incubated for 24 h with the recombinant Nef protein (~5 *μ*g/ml). The splenocytes (effector cells: E) were added to the target cells at an E : T ratio of 100 : 1. The effector and target cells were cocultured in complete RPMI-1640 supplemented with 10% heat-inactivated FBS and incubated at 5% CO_2_ and 37°C for 6 h under humidified conditions. Finally, following the manufacturer's instruction, the microplates were centrifuged at 250 × g and 4°C for five minutes, and the supernatants were harvested; this protocol was used to calculate the concentration of Granzyme B by ELISA (eBioscience).

### 2.10. Statistical Analysis

A one-way ANOVA (GraphPad Prism, GraphPad Software) was used for assessing discrepancies among test and control groups in immunological studies. The percentage of transfection was analyzed by flow cytometry using Student's *t*-test. Statistically, *p* < 0.05 indicates that the differences are significant. For each set of samples, all the parameters were expressed as mean ± standard deviation (SD). The experiments were performed in two independent experiments.

## 3. Results

### 3.1. Generation of the Recombinant Nef DNA and Protein

**Figure 3:**
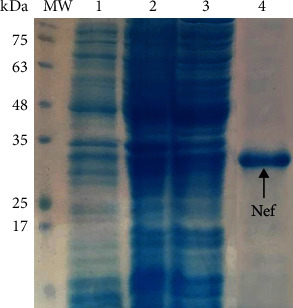
Expression and purification of HIV-1 Nef protein in *Rosetta*: lane 1: before induction; lane 2: 5 hours after induction with IPTG (1 mM); lane 3: 16 hours after induction with IPTG (1 mM); lane 4: the purified Nef protein; MW: molecular weight marker (prestained protein ladder, 10-180 kDa, Fermentas).

The purified HIV-1 Nef protein migrated as a clear band of ~27 kDa in SDS-PAGE as interpreted from our data (Figure 3). The concentration of recombinant protein ranged between 0.5 and 0.7 mg/ml. Also, the enzyme digestion of the recombinant pEGFP-Nef construct confirmed a clear band of ~648 bp for the Nef gene on an agarose gel.

### 3.2. Identification of DCs and MSCs

The flow cytometry analysis was used to identify MSCs and DCs by surface markers, as previously reported in our studies [19]. The percentages of positive (CD90.2) and negative (CD45) surface markers for MSCs were 99.96% and 0.43%, respectively. Moreover, the percentages of surface markers including CD11c, MHCII, CD86, and CD40 for DCs were 60.4%, 63.2%, 45%, and 39.2%, respectively.

### 3.3. Nef-GFP and GFP Expression under Mechanical and Chemical Treatments in DCs

**Figure 4:**
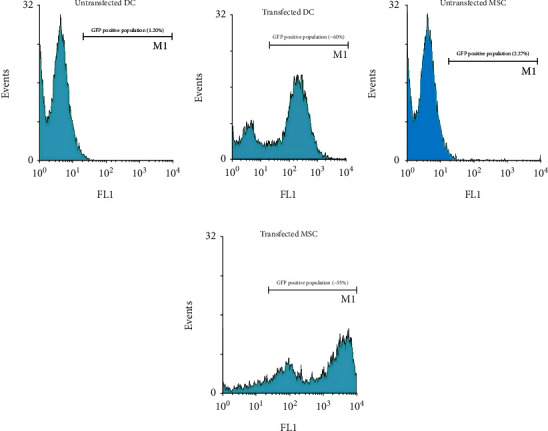
Transfection rates of DCs (b) and MSCs (d) with pEGFP-Nef using both Lipofectamine and equiaxial cyclic methods. Untransfected DCs (a) and MSCs (c) were shown as a negative control.

Considerably low transfection rate of pEGFP-N1 and pEGFP-Nef using both TurboFect™ and Lipofectamine transfection reagents into DCs was concluded from the flow cytometry results; the percentage of GFP expression was equal to 13.03 ± 0.18, and the percentage of Nef-GFP expression was equal to 11.21 ± 0.23. Moreover, the effects of equiaxial cyclic stretch alone were investigated. In this test, loading parameters were determined using cell viability and transfection efficacy. Mechanical loading conditions were as follows: loading time of 30 minutes, strain rates of 10% and 5%, and frequency 1 Hz. According to the results, because of the equiaxial cyclic stretch, the transfection rate of pEGFP-Nef and pEGFP-N1 into DCs was 15.04 ± 0.08 and 17.18 ± 0.19 for strain 10%, respectively. The used strain 5% showed 8-10% efficiency, which was lower than strain 10%. Thus, we selected strain 10%. The transfection efficiency of pEGFP-Nef and pEGFP-N1 into DCs, respectively, was increased up to 22.24 ± 0.13% and 25.15 ± 0.21% by the combination of TurboFect™ transfection reagent and equiaxial cyclic stretch loading. In addition, the expression percentage of GFP and Nef-GFP by the simultaneous use of Lipofectamine and equiaxial cyclic stretch was 44.11 ± 0.01 and 42.80 ± 0.11, respectively, indicating the high efficiency of Lipofectamine compared to TurboFect. These transfection rates were increased when we used chemical transfection with Lipofectamine reagent two times at 48 h intervals. These percentages were 68.07 ± 0.05 and 61.18 ± 0.03 for PEGFP-N1 and pEGFP-Nef transfection into DCs, respectively (Figure 4). The transfection results for gene expression under mechanical and chemical treatments in DCs are summarized in Table 2.

### 3.4. Nef-GFP and GFP Expression under Mechanical and Chemical Treatments in MSCs

Considerably low transfection rate of pEGFP-N1 and pEGFP-Nef using Lipofectamine and TurboFect™ transfection reagents into MSCs was concluded from the flow cytometry results; the percentage of GFP expression was equal to 10.23 ± 0.08, and the percentage of Nef-GFP expression was equal to 8.01 ± 0.43. Moreover, the effects of equiaxial cyclic stretch were investigated. In this test, the loading parameters were picked. The mechanical loading conditions were as follows: loading time of 15 minutes, strain rates of 10% and 5%, and frequency of 1 Hz. According to the results, because of the equiaxial cyclic stretch, the transfection rate of pEGFP-Nef and pEGFP-N1 into MSCs was 10.74 ± 0.28 and 12.80 ± 0.35 for strain 10%, respectively. The transfection rate was low for strain 5% (6-7%); thus, we selected strain 10%. The transfection efficiency of pEGFP-Nef and pEGFP-N1 into MSCs, respectively, increased up to 17.04 ± 0.20% and 20.05 ± 0.31% by the combination of TurboFect™ transfection reagent and equiaxial cyclic stretch loading. In addition, the expression rate of GFP and Nef-GFP by the simultaneous use of Lipofectamine and equiaxial cyclic stretch was 42.01 ± 0.11 and 40.4 ± 0.21, respectively, indicating the high efficiency of Lipofectamine compared to TurboFect. These transfection rates were increased when we used chemical transfection with Lipofectamine reagent two times at 48 h intervals. These percentages were 55.14 ± 0.23 and 62.28 ± 0.19 for pEGFP-Nef PEGFP-N1 transfection into MSCs, respectively (Figure 4). The transfection results for gene expression under mechanical and chemical treatments in MSCs are summarized in Table 3.

### 3.5. Antibody Responses

**Figure 5:**
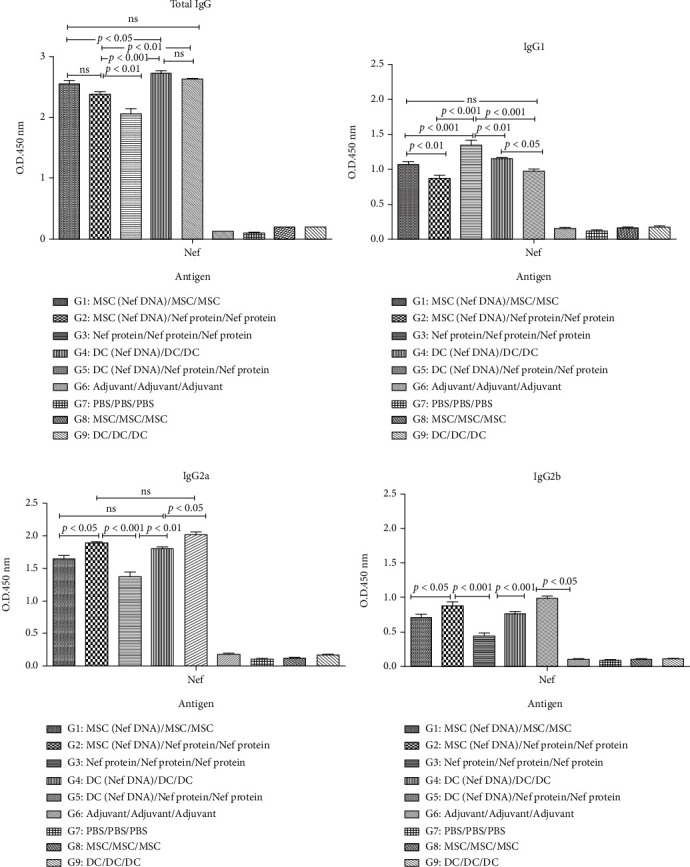
Antibody responses (total IgG: (a), IgG1: (b), IgG2a: (c), and IgG2b: (d)) against rNef protein in different regimens: all analyses were performed in duplicate for each sample. The results were shown as mean absorbance at 450 nm ± SD.

According to our data, the significant higher levels of total IgG and IgG1 in the sera of mice immunized by DCs and MSCs transfected with Nef DNA as heterologous (G2, G5) and homologous (G1, G4) regimens were reported compared to the group which received the recombinant Nef protein (G3) and control groups (*p* < 0.05, Figures 5(a) and 5(b)). Furthermore, significantly higher levels of IgG2b and IgG2a in the sera of mice immunized by MSCs and DCs transfected with pEGFP-Nef as heterologous regimen (G2, G5) were reported compared to other groups (*p* < 0.05, Figures 5(c) and 5(d)). Any significant anti-Nef antibody responses were not observed in the sera of control groups.

### 3.6. Secretion of Cytokines and Granzyme B

**Figure 6:**
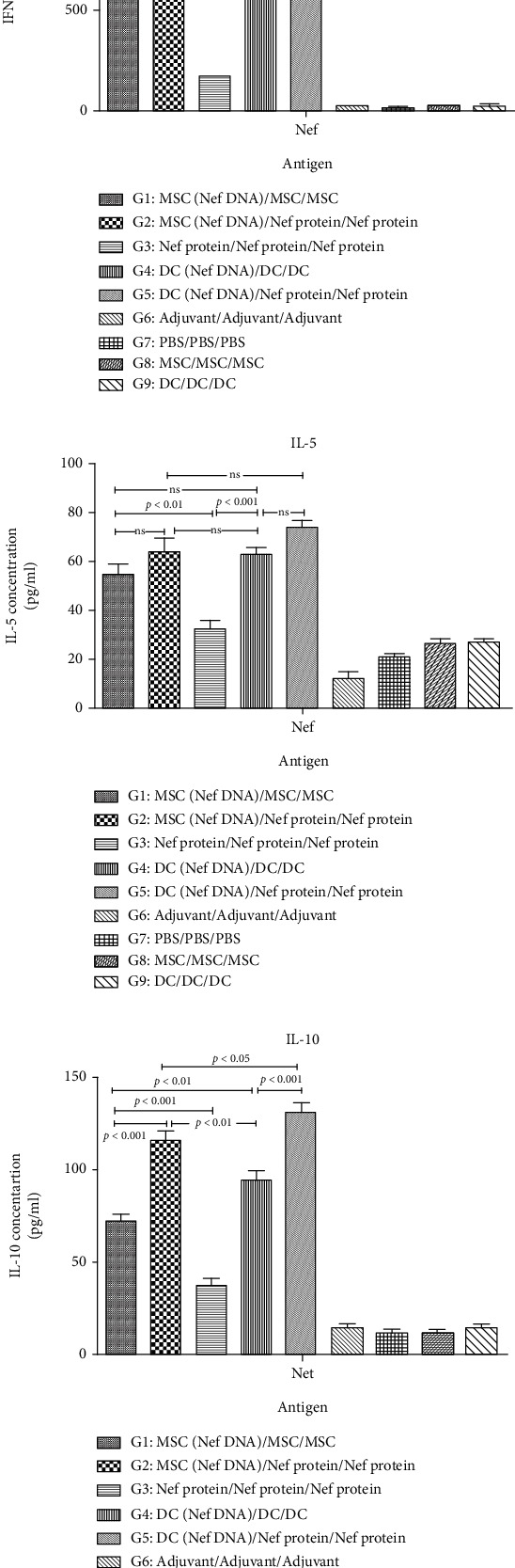
Secretion of IFN-*γ*, IL-5, and IL-10 in immunized groups with different formulations: the levels of IFN-*γ* (a), IL-5 (b), and IL-10 (c) were determined by ELISA as mean absorbance at 450 nm ± SD for each set of samples. All analyses were performed in duplicate for each sample.

**Figure 7:**
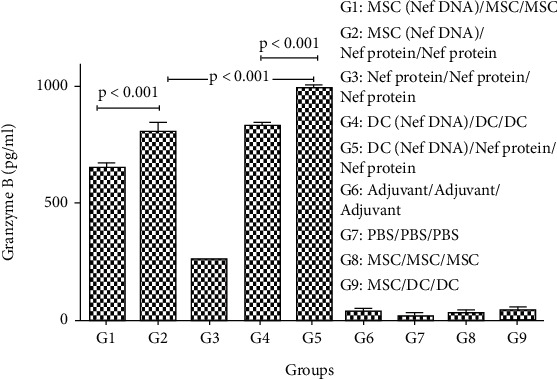
Granzyme B secretion in immunized groups with different regimens: the level of Granzyme B was assessed by ELISA as mean absorbance at 450 nm ± SD for each set of samples. All analyses were performed in duplicate for each sample.

The level of Nef-specific IFN-*γ* secretion in the groups immunized by MSCs as well as DCs transfected with pEGFP-Nef as a heterologous regimen (G2, G5) was observed to be significantly higher than that in other groups (*p* < 0.05, Figure 6(a)). Indeed, the IFN-*γ* secretion in all groups immunized by DCs as well as MSCs transfected with pEGFP-Nef as heterologous (G2, G5) and homologous (G1, G4) regimens was significantly higher compared to the group immunized with rNef protein (G3) and control groups (*p* < 0.05, Figure 6(a)). Besides, a higher generation of IL-5 by the groups immunized with homologous DCs and MSCs was reported compared to other groups. The IFN-gamma/IL-5 ratio has been meaningfully higher in all groups compared to control groups (Figure 6(b)). Furthermore, significantly higher secretion of IL-10 in the splenocytes restimulated with rNef protein was observed in the groups immunized by DCs transfected with Nef DNA (G5) and then MSCs transfected with Nef DNA (G2) as heterologous regimens compared to other groups (*p* < 0.05, Figure 6(c)). It was shown that the induction of IL-10 secretion was significantly higher in the group immunized by modified DCs and MSCs with pEGFP-Nef in comparison with the group immunized with Nef protein (G3, *p* < 0.05). However, a significant level of IFN-gamma compared to IL-10 was obtained in all test groups. Therefore, it was indicated that Nef is capable of significantly inducing the Th1 response in all regimens. According to the results of Granzyme B secretion in each group, the production of higher concentrations of Granzyme B by the groups immunized by MSCs as well as DCs transfected with Nef DNA as heterologous regimens (G2, G5) was significantly higher compared to other groups (*p* < 0.05, Figure 7). Moreover, the higher concentrations of Granzyme B were significantly secreted by the groups immunized by modified DCs and MSCs with pEGFP-Nef compared to the group immunized with rNef protein (G3, *p* < 0.05).

## 4. Discussion

Significant efforts have been performed to improve cell-based HIV vaccines. These types of vaccines can effectively stimulate the T-cell immune responses. The studies indicated that animals immunized with DCs loaded with envelope glycoproteins, HIV-1 viral lysate, or inactivated virus enhanced a potent immune response against HIV-1 infection [24]. Up to now, the 13 published clinical trials of DC-based HIV immunotherapy could induce effective immunological responses without side effects (e.g., autoimmunity), but only five of them showed antiviral responses. Thus, novel designed immunogens to pulse myeloid-derived DCs (MD-DCs) were used to improve vaccine efficiency [24, 25].

In the present research, the candidate antigen was HIV-1 Nef protein. We assessed and compared immune responses elicited by DCs and MSCs transfected with HIV-1 Nef in BALB/c mice. As known, most standard transfection approaches do not show a suitable efficiency for delivery of plasmid DNAs into MSCs and DCs; therefore, improvement of their potency is needed at present. We indicated that the efficiency of Nef DNA transfection was increased by the simultaneous use of mechanical and chemical vehicles. Indeed, chemical and mechanical methods showed low transfection efficacy for pEGFP-N1 and pEGFP-Nef plasmids, individually; but, their combination could enhance the transfection rates. Moreover, the use of Lipofectamine along with the mechanical method was significantly more effective than TurboFect associated with the mechanical method. Also, for the improvement of Nef expression, we transfected the cells with the chemical method two times at 48 h intervals. The efficiency of repeated transfection was significantly increased to use *in vivo*.

In a study, the delivery of *in vitro* RNA and DNA into monocyte-derived human DCs was evaluated by several nonviral transfection methods, including electroporation and lipofection. Delivery of green fluorescent protein (GFP) DNA was 10-11% using both delivery systems. Delivery GFP RNA was 11% and 20% using electroporation and lipofection, respectively [26]. Our data showed higher efficiency of chemical and mechanical methods as compared to these techniques. In another study, an optimized commercial protocol was used for high-throughput transfection of human primary DCs using electroporation, with low cell toxicity and a lack of DC maturation (CD86 and MHCII). The cell viability and transfection efficiency of GFP DNA were more than 70% and 50%, respectively [27]. This improved electroporation showed higher efficiency for DC transfection with DNA and almost similar to our results using the combination of Lipofectamine and mechanical methods. However, twice transfection in our study increased the expression more than the improved electroporation. Previously, an approach on the basis of repeated transfections at the 48 h interval for 168 hours has been presented. *In vivo* data also showed the capability of this method in enhancing transfection versus single administration [2]. Moreover, a novel extended gene expression (EGE) method has been applied using the combination of medium exchange and repeated transfection of cell cultures with the recombinant plasmid DNA. According to the obtained data, the prolongation of the production period was in the range of 192 and 240 hours using the EGE strategy [28].

On the other hand, it was shown that mouse DCs and MSCs transfected with Nef DNA as heterologous and homologous could stimulate IFN-gamma (a Th1 cytokine) and activate Granzyme B compared to rNef protein immunization. Also, Nef-specific IgG2a and IgG2b antibodies were produced by B cells stimulated with modified DCs and MSCs in comparison with other groups. In DCs and then MSCs transfected with Nef DNA as heterologous DC or MSC prime/protein boost, the immune responses were significantly more than those in other groups. Indeed, Nef DNA-transfected MSCs or Nef DNA-transfected DCs were capable of directing the immune responses from Th2-dominance towards Th1, leading to reduced IgG1 production. A study showed that human DCs transfected with allergen-DNA could shift the human allergic immune responses from Th2-dominance towards Th1 and Tc1, which results in increased IgG4 production and decreased IgE, and activating IFN-c-producing CD8^+^ T-cells [29]. Also, mononuclear cell (MNC) antitumor activity was enhanced by DNA-transfected DCs. A cytotoxic response was elicited by the DNA-transfected DCs that its efficiency was as same as that of tumor lysate-loaded DCs. The data suggested that the transfected DCs can be used for inducing an antitumor immune response in colorectal MNCs [30]. Moreover, untreated patients with chronic HIV-1 infection were vaccinated by three doses of autologous MD-DCs transfected with heat-inactivated autologous HIV-1, which was shown as a safe, well-tolerated, and feasible treatment. It was observed that vaccine recipients had a modest significant reduction in their plasma viral load compared to control groups [25]. In this line, a therapeutic HIV-1 vaccine based on DCs loaded with apoptotic bodies could induce T-cell activation and cytolysis in phase I/II clinical trial without severe side effects [6].

Some studies showed that MSC strategies overcome problems caused by DNA-based and DC-based vaccines [5]. A study showed that efficient tools for *ex vivo* modification of human MSCs were provided by lentivirus pseudotypes bearing Env glucoproteins [4]. Another study indicated the higher effectiveness of MSCs compared to adenovirus as a cytokine gene delivery system. For instance, the intradermal injection of MSCs/ IL-12M was an excellent method for stimulating high levels of tumor-specific T-cell responses and subsequently inhibited solid tumor growth [31]. Herein, we used MSCs for the delivery of HIV-1 Nef antigen by improving transfection efficiency and evaluating immune responses. The results showed that the modified MSCs have similar efficiency with the modified DCs in stimulating immune responses. Although heterologous Nef DC prime/protein boost showed higher efficacy in the production of IFN-*γ*, Granzyme B, IgG2b, and IgG2a *in vivo*, these responses in both heterologous and homologous MSC regimens were significantly higher than protein strategy (Nef protein prime/Nef protein boost). These results were similar to our previous studies on the potency of DCs and MSCs transfected with HIV-1 MPER-V3 DNA in heterologous DC or MSC prime/peptide boost immunizations [32]. Herein, we showed that improvement of delivery systems (i.e., combination of mechanical and chemical methods) could influence the potency of modified DCs and MSCs with Nef DNA followed by the recombinant Nef protein regimens as observed for the transfected DCs and MSCs with MPER-V3 DNA followed by MPER-V3 peptide. However, HIV-1 Nef protein is important as an antigen candidate for HIV-1 therapeutic vaccine as compared to other HIV antigens.

In conclusion, the results showed that the modified MSC- and DC-based immunizations with Nef antigen would be excellent approaches for the induction of strong immune responses without the use of any additional adjuvant against HIV-1 infections.

## Figures and Tables

**Table 1:** Different immunization strategies using MSCs, DCs, and proteins in BALB/c mice.

Group	Modality	First injection	Second injection	Third injection
G1	MSC/MSC/MSC	MSC (Nef DNA) (2 × 10^5^ cells)	MSC (Nef DNA) (2 × 10^5^ cells)	MSC (Nef DNA) (2 × 10^5^ cells)
G2	MSC/protein/protein	MSC (Nef DNA) (2 × 10^5^ cells)	Nef protein+ Montanide (10 *μ*g)	Nef protein+ Montanide (10 *μ*g)
G3	Protein/protein/protein	Nef protein+ Montanide (10 *μ*g)	Nef protein+ Montanide (10 *μ*g)	Nef protein+ Montanide (10 *μ*g)
G4	DC/DC/DC	DC (Nef DNA) (1 × 10^6^ cells)	DC (Nef DNA) (1 × 10^6^ cells)	DC (Nef DNA) (1 × 10^6^ cells)
G5	DC/protein/protein	DC (Nef DNA) (1 × 10^6^ cells)	Nef protein+ Montanide (10 *μ*g)	Nef protein+ Montanide (10 *μ*g)
G6	Control	Montanide	Montanide	Montanide
G7	Control	PBS	PBS	PBS
G8	Control	MSC	MSC	MSC
G9	Control	DC	DC	DC

**Table 2:** Flow cytometry analysis of gene expression under mechanical and chemical treatments in DCs.

Methods	Percentage
GFP expression using both TurboFect™ and Lipofectamine transfection reagents	13.03 ± 0.18
pEGFP-Nef expression using both TurboFect™ and Lipofectamine transfection reagents	11.21 ± 0.23
pEGFP-Nef expression using equiaxial cyclic stretch (30 minutes, strain rates of 10%, and frequency 1 Hz)	15.04 ± 0.08
pEGFP-N1 expression using equiaxial cyclic stretch (30 minutes, strain rates of 10%, and frequency 1 Hz)	17.18 ± 0.19
pEGFP-N1 and pEGFP-Nef expression using equiaxial cyclic stretch (30 minutes, strain rates of 5%, and frequency 1 Hz)	8-10%
pEGFP-Nef expression using a combination of TurboFect™ transfection reagent and equiaxial cyclic stretch (30 minutes, strain rates of 10%, and frequency 1 Hz)	22.24 ± 0.13%
pEGFP-N1 expression using a combination of TurboFect™ transfection reagent and equiaxial cyclic stretch (30 minutes, strain rates of 5%, and frequency 1 Hz)	25.15 ± 0.21%
pEGFP-Nef expression using a combination of TurboFect™ transfection reagent and equiaxial cyclic stretch (30 minutes, strain rates of 10%, and frequency 1 Hz)	44.11 ± 0.01
pEGFP-N1 expression using a combination of Lipofectamine transfection reagent and equiaxial cyclic stretch (30 minutes, strain rates of 5%, and frequency 1 Hz)	42.80 ± 0.11
pEGFP-Nef expression using a combination of Lipofectamine transfection reagent two times at 48 h intervals and equiaxial cyclic stretch (30 minutes, strain rates of 10%, and frequency 1 Hz)	68.07 ± 0.05
pEGFP-Nef expression using a combination of Lipofectamine transfection reagent two times at 48 h intervals and equiaxial cyclic stretch (30 minutes, strain rates of 10%, and frequency 1 Hz)	61.18 ± 0.03

**Table 3:** Flow cytometry analysis of gene expression under mechanical and chemical treatments in MSCs.

Methods	Percentage
GFP expression using both TurboFect™ and Lipofectamine transfection reagents	10.23 ± 0.08
pEGFP-Nef expression using both TurboFect™ and Lipofectamine transfection reagents	8.01 ± 0.43
pEGFP-Nef expression using equiaxial cyclic stretch (30 minutes, strain rates of 10%, and frequency 1 Hz)	10.74 ± 0.28
pEGFP-N1 expression using equiaxial cyclic stretch (30 minutes, strain rates of 10%, and frequency 1 Hz)	12.80 ± 0.35
pEGFP-N1 and pEGFP-Nef expression using equiaxial cyclic stretch (30 minutes, strain rates of 5%, and frequency 1 Hz)	6-7%
pEGFP-Nef expression using a combination of TurboFect™ transfection reagent and equiaxial cyclic stretch (30 minutes, strain rates of 10%, and frequency 1 Hz)	17.04 ± 0.20%
pEGFP-N1 expression using a combination of TurboFect™ transfection reagent and equiaxial cyclic stretch (30 minutes, strain rates of 5%, and frequency 1 Hz)	20.05 ± 0.31%
pEGFP-Nef expression using a combination of TurboFect™ transfection reagent and equiaxial cyclic stretch (30 minutes, strain rates of 10%, and frequency 1 Hz)	42.01 ± 0.11
pEGFP-N1 expression using a combination of Lipofectamine transfection reagent and equiaxial cyclic stretch (30 minutes, strain rates of 5%, and frequency 1 Hz)	40.4 ± 0.21
pEGFP-Nef expression using a combination of Lipofectamine transfection reagent two times at 48 h intervals and equiaxial cyclic stretch (30 minutes, strain rates of 10%, and frequency 1 Hz)	55.14 ± 0.23
pEGFP-Nef expression using a combination of Lipofectamine transfection reagent two times at 48 h intervals and equiaxial cyclic stretch (30 minutes, strain rates of 10%, and frequency 1 Hz)	62.28 ± 0.19

## Data Availability

All data are available in the submitted manuscript.
