# Erratum to “Development of Delivery Systems Enhances the Potency of Cell-Based HIV-1 Therapeutic Vaccine Candidates”

**DOI:** 10.1155/2021/9763540

**Published:** 2021-07-07

**Authors:** Amin Hadi, Abbas Rastgoo, Maryam Eskandarian, Nooshin Haghighipour, Azam Bolhassani

**Affiliations:** ^1^Cellular and Molecular Research Center, Yasuj University of Medical Sciences, Yasuj, Iran; ^2^School of Mechanical Engineering, University of Tehran, Tehran, Iran; ^3^Department of Immunology, Tarbiat Modares University, Tehran, Iran; ^4^National Cell Bank of Iran, Pasteur Institute of Iran, Tehran, Iran; ^5^Department of Hepatitis and AIDs, Pasteur Institute of Iran, Tehran, Iran

In the article titled “Development of Delivery Systems Enhances the Potency of Cell-Based HIV-1 Therapeutic Vaccine Candidates” [1], the contact details for author “Azam Bolhassani” were incorrect. The correct contact information is a_bolhasani@pasteur.ac.ir “a_bolhasani@pasteur.ac.ir.”

The error was introduced during the production process of the article, and Hindawi apologises for causing this error in the article.
